# The effects of combined cognitive training on prospective memory in older adults with mild cognitive impairment

**DOI:** 10.1038/s41598-021-95126-z

**Published:** 2021-08-02

**Authors:** Yifan Chen, Wei Zhou, Zijing Hong, Rongrong Hu, Zhibin Guo, Shen Liu, Lin Zhang

**Affiliations:** 1 Department and Institute of Psychology, Ningbo University, No. 616 Fenghua Road, Jiangbei District, Ningbo, 315211 China; 2 School of Management, Jinan University, Guangzhou, China; 3 School of Humanities and Social Sciences, University of Science and Technology of China, No. 96 Jinzhai Road, Baohe District, Hefei, 230022 China

**Keywords:** Psychology, Biomarkers

## Abstract

This study aimed to assess the effects of combined cognitive training on prospective memory ability of older adults with mild cognitive impairment (MCI). A total of 113 participants were divided into a control group and three intervention groups. Over three months, the control group received only community education without any training, whereas for the first six weeks, an executive function training group received executive function training, a memory strategy training group received semantic encoding strategy training, and the combined cognitive training group received executive function training twice a week for the first six weeks, and semantic encoding strategy training twice a week for the next six weeks. The combined cognitive training group showed improvement on the objective neuropsychological testing (Montreal Cognitive Assessment scale). The memory strategy training group showed improvement on the self-evaluation scales (PRMQ-PM). Combined cognitive training improved the prospective memory and cognitive function of older adults with MCI.

Alzheimer’s disease (AD), a chronic disease in older adults, is the “fourth leading cause of death,” followed by cardiovascular disease, cerebrovascular disease, and cancer; however, its cause remains unknown. According to the World Alzheimer Report 2018, released by Alzheimer’s Disease International, approximately 50 million people were diagnosed with AD worldwide by the end of 2018, and the numbers of patients with AD is estimated to reach 152 million by 2050^1^. The prodromal phase of AD is mild cognitive impairment (MCI), expressed as a decline in cognitive function^2^. AD is an irreversible degenerative disease of the central nervous system; therefore, it is critical to address its MCI phase, especially as studies have highlighted that timely cognitive intervention in older adults with MCI can delay the decline in their cognitive abilities^3,4^.

Older adults with a loss of prospective memory are at a high risk for AD and cognitive training can effectively improve their cognitive function^5^. Prospective memory, refers to the memory of planned activities and events at a certain moment in the future. Studies have found that both these aspects in older adults with MCI or AD undergo differing degrees of impairment^6^, but that prospective memory impairment in older adults with MCI is less severe than for those with AD^7^. Studies conducted on patients with dementia have found that prospective memory performance may play a role in predicting AD. Studies based on clinical scales^8^, laboratory tasks^6,9,10^, natural tasks^10^, and questionnaires^11^ have found that older adults with MCI have worse prospective memory performance than healthy older adults, and that they tend to experience more prospective memory failures in their daily lives^11^. These may even cause the inability to look after oneself. Therefore, interventions concerning prospective memory are essential for older adults with MCI to improve their quality of life.

Studies have shown that older adults’ cognitive function can be improved through appropriate training^5,12^, as this changes their brain capacity and brain structure, indicating that cognition in older adults is plastic^13^. The current mainstream explanation for the decline of cognitive function in older adults is the “decline hypothesis,” which holds that mainly, the decline in executive function causes the decline in cognitive function^14^; however, previous investigations concerning training cognitive function and neural mechanisms have been based on healthy older adults. Older adults with MCI suffer a relatively more severe decline of cognitive function, so it is more important to conduct studies with them.

Executive function is the most advanced cognitive activity in the brain, with the frontal lobe (especially the prefrontal lobe) being the key physiological organ for it. Compared with other brain regions, the function of the frontal lobe of the elderly declines faster^15^ and has stronger plasticity^16^; however, there are only a few studies on executive neuroplasticity in older adults with MCI. If the cognitive abilities of these older adults can be improved through executive function training, this would indicate that such adults retained plasticity in executive function.

Most previous interventions involving individuals with MCI have used single memory training or executive function training such as memory strategy and working memory training^3,17^. This single memory training approach has many limitations. First, there is a limited transfer effect in single memory training—improvement rarely transfers to other cognitive functions^18,19^. Second, even after training, older adults still have difficulty utilizing memory strategies—some studies have found that differences after memory training increased^20^. This may be due to the decline in age-related executive function preventing older adults from effectively applying memory strategies to memory tasks^21^.

In addition, a decline in prospective memory in older adults with MCI has been found to be related to executive function as well^22^. To complete a prospective memory task, individuals need to monitor the clues prompting the intention to undertake the task, set aside or inhibit any ongoing task at the appropriate time, and shift attention to the prospective memory task. This process is largely dependent on executive functions^23^. However, compared with healthy older adults, those with MCI are worse at task conversion processing, coping with complex memory tasks, and setting aside ongoing tasks, which may be due to a decline in executive function.

Studies have indicated that conducting executive function training before memory strategy training may promote the application of memory strategies^5^; however, it remains unclear whether this method would effectively improve prospective memory abilities. Cheng et al.^24^ found that combined cognitive training could effectively improve the cognitive function of older adults with MCI; however, the intervention measures adopted were calligraphy and painting and no unified intervention method was applied. Further, prospective memory in older adults with MCI was not examined.

Compared to single cognitive training, combined cognitive training involves complex cognitive processes, which have a greater impact on multiple cognitive functions and a greater transfer effect^24–26^. Some studies have conducted interventions involving card games among healthy older adults to combine training with social interactions. The results showed that this training method significantly improved cognitive function, and that the training effect was transferred to untrained test tasks^12^. Some studies have combined transcranial direct current stimulation with working memory training and the results indicated that this training method not only had a better training effect on working memory than single training, but also transferred training effects to similar untrained memory tasks^27^. Therefore, it would appear that combined training has a greater training effect and greater transfer effect than single training.

Based on these considerations, this study proposed the following hypotheses:

## Hypothesis 1

Combined cognitive training has a stronger near-transfer effect on prospective memory than single memory and single executive function training.

## Hypothesis 2

Compared with single training, combined cognitive training does not only have a better near-transfer effect on prospective memory but also has a better far-transfer effect on cognitive ability and other related aspects; it maintains training benefits more effectively.

To the best of our knowledge, no study on targeted training in terms of combined cognitive training related to prospective memory has been conducted. Therefore, this study adopted a training method that combined executive function training and memory strategy training to explore the training benefits for prospective memory and the transfer effect. Since executive function impairment may be the main cause of prospective memory failure in individuals with MCI^22^, executive function training was conducted before memory training. Cognitive function and the daily living abilities of older adults with MCI are reduced compared with healthy elderly individuals, but it may be improved in older adults with MCI through cognitive training^5^. Therefore, this study aimed to determine whether cognitive function and the prospective memory of older adults with MCI could be enhanced through cognitive training in an effort to improve their quality of life (Table 1).

**Table 1:** Characteristics of the control group and intervention groups.

Item	Category	Control group (*n* = 33)	Executive function training group (*n* = 27)	Memory strategy training group (*n* = 26)	Combined training group (*n* = 27)	*p*
**Gender**	Male	15 (45.5)	13 (48.1)	11 (42.3)	15 (55.6)	0.798
	Female	18 (54.5)	14 (51.9)	16 (57.7)	12 (44.4)	
**Age**		70.39 (7.36)	73.67 (7.51)	73.73 (5.83)	72.78 (7.82)	0.233
**Education level**	Primary school or below	7 (21.2)	5 (18.5)	10 (38.5)	8 (29.6)	0.452
	Junior school	18 (54.5)	13 (48.1)	9 (34.6)	14 (51.9)	
	High school	3 (9.1)	4 (14.8)	7 (26.9)	1 (3.7)	
	Junior college	4 (12.1)	5 (18.5)	0 (0.0)	3 (11.1)	
	Undergraduate	1 (3.0)	0 (0.0)	0 (0.0)	1 (3.7)	

Numbers are outside the brackets, percentages are inside the brackets.

## Results

### Pre-test results

A single factor analysis of variance (ANOVA) was performed on the Montreal Cognitive Assessment Scale (MoCA), Instrumental Activities of Daily Living Scale (IADL), Prospective and Retrospective Memory Questionnaire-Prospective Memory (PRMQ-PM), and Virtual Week (VW) task scores of the four groups before beginning the experiment. The results are shown in Table 2. The scores on the MoCA (*F* [3, 109] = 0.61, *p* = 0.611), IADL (*F* [3, 109] = 1.33, *p* = 0.269), PRMQ-PM (*F* [3, 109] = 0.86, *p* = 0.464), and VW (*F* [3, 109] = 0.23, *p* = 0.874) did not vary significantly. This indicated that the intervention and control groups were similar.

**Table 2:** Baseline levels of the participants before intervention (mean [*M*] ± standard deviation [*SD*]).

Measure	Control group (*n* = 33)	Executive function training group (*n* = 27)	Memory strategy training group (*n* = 26)	Combined cognitive training group (*n* = 27)
MoCA	15.18 ± 2.16	15.37 ± 1.84	15.35 ± 1.57	15.85 ± 2.20
IADL	15.58 ± 2.57	15.26 ± 2.28	14.62 ± 3.11	16.19 ± 3.64
PRMQ-PM	25.09 ± 5.26	25.00 ± 5.46	23.31 ± 5.31	23.78 ± 4.15
VW	4.14 ± 1.30	3.89 ± 1.32	3.96 ± 1.63	4.08 ± 0.68

*MoCA* montreal cognitive assessment scale, *IADL* instrumental activities of daily living scale, *PRMQ-PM* prospective and retrospective memory questionnaire-prospective memory, *VW* virtual week task.

### Post-test results

#### Retrospective memory questionnaire-prospective memory (PRMQ-PM) scores

The PRMQ-PM scores before and after training are presented in Table 3. We performed a four-group (control group/executive function training group/memory strategy training group/combined cognitive training group) × 2 test time (pre-test/post-test) variance analysis of repeated measures. The results showed that the overall group effect was not significant *(F* [3, 109] = 0.36, *p* = 0.785), however, the overall test time effect (*F* [1, 109] = 73.18, *p* < 0.001, η^2^_*p*_ = 0.402), and the interaction (*F* [3, 109] = 11.46, *p* < 0.001, η^2^_*p*_ = 0.240) were statistically significant. Further multiple comparisons showed that compared with their pre-test PRMQ-PM scores, the post-test scores of the executive function training group (*p* < 0.001), the memory strategy training group (*p* < 0.001), and the combined cognitive training group (*p* < 0.001) all improved significantly, while the control group’s scores for the two tests did not vary significantly (*p* = 1.000). To compare the training effect on the cognitive ability of the participants in the three training groups, a one-way analysis of variance was performed on the training effect of the PRMQ-PM. The results showed that the overall effect on the training groups was significant (*F* [2, 79] = 4.83, *p* < 0.05, η^2^_*p*_ = 0.111). Further multiple comparison showed that the memory strategy training group showed a significantly larger training effect, as defined by the difference between baseline and post-test (*p* < 0.05), and that the scores of the latter two groups were not significantly different (*p* = 0.588).

**Table 3:** Differences between prospective memory in the prospective and retrospective memory questionnaire-prospective memory (PRMQ-PM) scores before and after intervention (mean [*M*] ± standard deviation [*SD*]).

Score	Control group (*n* = 33)	Executive function training group (*n* = 27)	Memory strategy training group (*n* = 26)	Combined cognitive training group (*n* = 27)
Pre-test	25.09 ± 5.26	25.00 ± 5.46	23.31 ± 5.31	23.78 ± 4.15
Post-test	25.09 ± 4.03	27.26 ± 4.76	27.58 ± 3.86	26.41 ± 3.04
Training effect	0 ± 3.51	2.26 ± 2.64	4.27 ± 2.54	2.63 ± 2.31

#### Virtual week (VW) task results

The scores for the VW task before and after training are shown in Table 4. We performed a four group (control group/executive function training group/memory strategy training group/combined cognitive training group) × 2 test time (pre-test/post-test) variance analysis of repeated measures. The results showed that the overall group effect was significant (*F* [3, 109] = 12.47, *p* < 0.001, η^2^_*p*_ = 0.256), and so were the overall test time effect (*F* [1, 109] = 202.47, *p* < 0.001, η^2^_*p*_ = 0.650) and interaction (*F* [3, 109] = 36.54, *p* < 0.001, η^2^_*p*_ = 0.501). Further multiple comparisons showed that compared with their pre-test VW task scores, the post-test scores of the executive function training group (*p* < 0.001), the memory strategy training group (*p* < 0.001), and the combined cognitive training group (*p* < 0.001) all significantly improved, while the scores of the control group for the two tests did not vary significantly (*p* = 0.166). To compare the training effect on participants’ prospective memory in the three training groups, a one-way analysis of variance was performed on the training effect of the VW task. The results showed that the overall effect on the training group was not significant (*F* [2, 79] = 2.43, *p* = 0.095).

**Table 4:** Differences between the scores of the virtual week tasks before and after intervention (mean [*M*] ± standard deviation [*SD*]).

Score	Control group (*n* = 33)	Executive function training group (*n* = 27)	Memory strategy training group (*n* = 26)	Combined cognitive training group (*n* = 27)
Pre-test	4.14 ± 1.30	3.89 ± 1.32	3.96 ± 1.63	4.08 ± 0.68
Post-test	3.84 ± 0.92	6.04 ± 1.20	6.06 ± 1.11	6.85 ± 0.84
Training effect	− 0.30 ± 1.27	2.15 ± 1.53	2.10 ± 1.17	2.77 ± 0.96

#### Montreal cognitive assessment scale (MoCA) results

The MoCA scores of the three groups before and after training are shown in Table 5. We performed a four group (control group/executive function training group/memory strategy training group/combined cognitive training group) × 2 test time (pre-test/post-test) variance analysis of repeated measures. The results showed that the overall group effect was significant (*F* [3, 109] = 4.90, *p* < 0.05, η^2^_*p*_ = 0.119), and so were the overall test time effect (*F* [1, 109] = 59.94, *p* < 0.001, η^2^_*p*_ = 0.360) and interaction (*F* [3, 109] = 12.36, *p* < 0.001, η^2^_*p*_ = 0.254). Further multiple comparisons showed that compared with their pre-test MoCA scores, the post-test scores of the executive function (*p* < 0.001), memory strategy (*p* < 0.001), and combined cognitive training groups (*p* < 0.001) all significantly improved, while control group’s scores for the two tests did not vary significantly (*p* = 0.415). One-way analysis of variance was performed on the training effect of the MoCA. The results showed that the overall group effect was significant (*F* [2, 79] = 13.09, *p* < 0.001, η^2^_*p*_ = 0.254). Further paired comparison showed a significantly improved training effect in the combined cognitive training group compared to the executive function and the memory strategy training groups (*p* < 0.001), and that the training effect on the latter two groups was not significantly different (*p* = 0.799).

**Table 5:** Differences between the montreal cognitive assessment scale (MoCA) scores before and after intervention (mean [*M*] ± standard deviation [*SD*]).

Score	Control group (*n* = 33)	Executive function training group (*n* = 27)	Memory strategy training group (*n* = 26)	Combined cognitive training group (*n* = 27)
Pre-test	15.18 ± 2.16	15.37 ± 1.84	15.35 ± 1.57	15.85 ± 2.20
Post-test	15.42 ± 3.26	16.26 ± .98	16.35 ± 2.21	18.70 ± 2.48
Training effect	0.24 ± 1.97	0.89 ± 1.42	1.00 ± 1.58	2.85 ± 1.73

#### Instrumental activities of daily living scale (IADL) results

The IADL scores of the three groups before and after training are shown in Table 6. We performed a four group (control group/executive function training group/memory strategy training group/combined cognitive training group) × 2 test time (pre-test/post-test) variance analysis of repeated measures. The results showed that the overall group effect was not significant (*F* [3, 109] = 1.09, *p* = 0.357), and so was the overall test time effect (*F* [1, 109] = 1.233, *p* = 0.269). The interaction between the groups and the test times was not significant (*F* [3, 109] = 0.065, *p* = 0.978).

**Table 6:** Differences between the instrumental activities of daily living scale (IADL) scores before and after intervention (mean [*M*] ± standard deviation [*SD*]).

Score	Control group (*n* = 33)	Executive function training group (*n* = 27)	Memory strategy training group (*n* = 26)	Combined cognitive training group (*n* = 27)
Pre-test	15.58 ± 2.57	15.26 ± 2.28	14.62 ± 3.11	16.19 ± 3.64
Post-test	15.18 ± 3.43	15.15 ± 2.37	14.35 ± 4.73	15.93 ± 4.54
Training effect	− 0.39 ± 2.82	− 0.11 ± 1.31	− 0.27 ± 2.70	− 0.26 ± 2.64

### Follow-up test results

**Figure 1:**
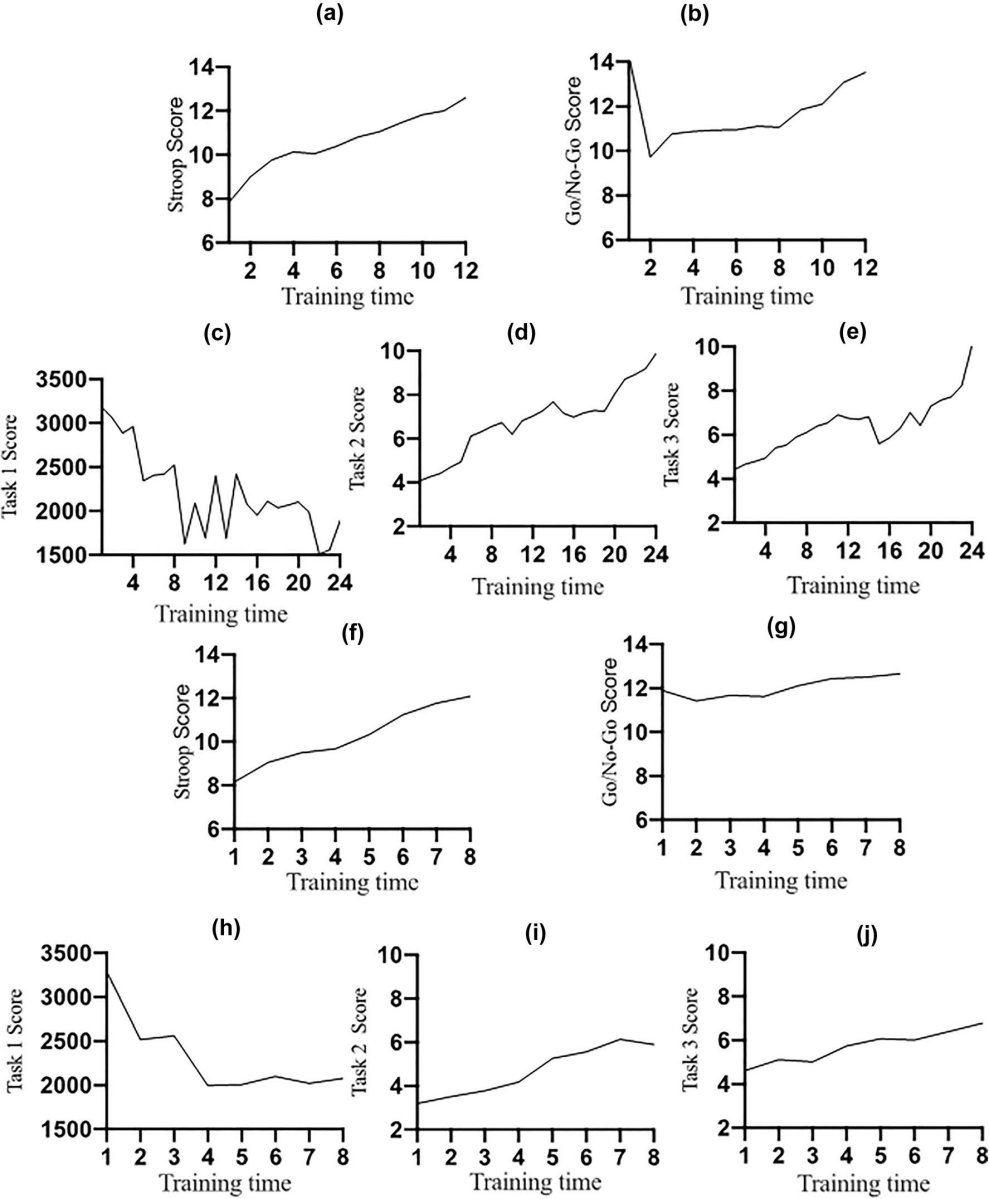
(**a**), (**b**) Scores of the Stroop (left)/Go/No-Go (right) tasks in the executive function training group. Task score = accuracy/average response time * 10,000. (**c**–**e**): Scores of the fine encoding task 1 (left)/task 2 (middle)/task 3 (right) in the memory strategy training group. The response time of task 1 was recorded as the score of task 1. The scores of task 2 and task 3 = accuracy/average response time *10,000. (**f**), (**g**): Scores of the Stroop (left)/Go/No-Go (right) tasks in the combined training group. (**h**–**j**): Scores of the fine encoding task 1 (left)/task 2 (middle)/task 3 (right) of the combined training group.

The follow-up data has been shown in Table 7. Table 8 shows the difference between the follow-up data and the pre-test results. We performed a three group (executive function training group/memory strategy training group/combined cognitive training group) × 2 test time (pre-test/follow up-test) variance analysis of repeated measures, the ANOVAs showed no significant group * time interactions on VW, MoCA, IADL(Table 8). But on PRMQ-PM, the results showed that the overall group effect was not significant (F [2, 77] = 0.218, *p* = 0.805), the interaction was significant (F [2, 77] = 3.626, *p* = 0.031). But further multiple comparison showed there is no significant difference between three groups. The above-mentioned results could be seen in Fig. 1.

**Table 7:** Follow-up results of the participants (mean [M] ± standard deviation [SD]).

Measure	Control group (*n* = 33)	Executive function training group (*n* = 27)	Memory strategy training group (*n* = 26)	Combined cognitive training group (*n* = 27)
MoCA	14.76 ± 1.80	16.11 ± 0.89	16.15 ± 2.39	18.11 ± 2.68
IADL	14.94 ± 2.52	14.59 ± 2.21	14.62 ± 3.45	14.85 ± 2.49
PRMQ-PM	23.58 ± 3.96	26.30 ± 4.71	26.73 ± 3.66	26.15 ± 2.98
VW	2.77 ± 0.76	4.36 ± 0.85	4.72 ± 0.90	4.99 ± 0.89

*MoCA* montreal cognitive assessment scale, *IADL* instrumental activities of daily living scale, *PRMQ-PM* prospective and retrospective memory questionnaire-prospective memory, *VW* virtual week task.

**Table 8:** Comparison of pre-test and follow-up difference between the three groups (mean [*M*] ± standard deviation [*SD*]).

Variable	Executive function training group	Memory strategy training group	Combined cognitive training group	*p*
MoCA	0.74 ± 1.32	0.81 ± 2.68	2.26 ± 3.40	0.062
IADL	− 0.67 ± 1.33	0 ± 1.90	− 1.33 ± 2.54	0.057
PRMQ-PM	1.30 ± 3.16	3.42 ± 2.77	2.37 ± 2.67	0.031
VW	0.47 ± 1.43	0.76 ± 1.37	0.91 ± 0.83	0.421

*MoCA* montreal cognitive assessment scale, *IADL* instrumental activities of daily living scale, *PRMQ-PM* prospective and retrospective memory questionnaire-prospective memory, *VW* virtual week task.

*P* for three group (executive function training group/memory strategy training group/combined cognitive training group) × 2 test time (pre-test/follow up-test) ANOVAs.

## Discussion

This study utilized executive function training, memory strategy training, and combined cognitive training of prospective memory and executive function to explore the near-transfer effect on prospective memory of older adults with MCI and the far-transfer effect on their cognitive and daily living abilities. The results showed that combined cognitive training could significantly improve the cognitive function of older adults with MCI but not significantly improve their daily living ability and that combined cognitive training could improve the prospective memory of older adults with MCI, but the training effect of prospective memory is not as good as the memory strategy training group.

Regarding near-transfer effect on prospective memory, the current study hypothesized that combined cognitive training would improve the prospective memory of older adults with MCI more than executive function training and memory strategy training; however, according to the results of the self-evaluation scales (PRMQ-PM) we found that memory strategy training had a stronger effect on training prospective memory, which did not support our hypothesis. This result may have been due to the insufficient training of executive functions. Current studies have shown that training duration is related to the improvement of executive function training^28^. Studies by Holmes et al.^29^ and Klingberg et al.^30,31^ reported that sufficient training time (at least 3 weeks or 8 h of working memory training) was an important factor in transferring the training effect to other tasks. However, in the current study, only 6 h of executive function training was conducted for the combined cognitive training group, which may have made it difficult for the participants to effectively improve their memory function. Greater improvement in prospective memory was found for the memory strategy training group. For the objective neuropsychological testing (VW task), there were no significant difference in the scores of the three groups, possibly because this task is complicated. Older adults tend to adopt strategic memory, such as remembering tasks that are easy or which contain special meanings and this leads to a floor effect in training results^32^.

Regarding the far-transfer effect on cognitive abilities, combined cognitive training showed a better training effect than memory strategy training and executive function training, which was consistent with previous research^24–26^. In the current study, through conducting executive function training for older adults with MCI, it was found that their cognitive function could be improved through training, which supports the understanding that older adults with MCI retain executive function plasticity. In terms of the transfer effect of executive function training, no obvious far-transfer effect was found to be retained from executive function training, which corresponds with previous studies^33^. Some studies have found that training “switch” sub-functions in executive functions can not only improve the inhibitory capacity of individuals, but also have a significant transfer effect on working memory and intelligence^34^. This inconsistency in the results concerning the transfer effect indicates that there are challenges in distinguishing various cognitive abilities in training and that the transfer effect can simultaneously vary^35^. Therefore, future research needs to explore which type of executive function training produces the greatest transfer effect. In terms of the maintenance of the training effect, the current study found that the training effect was well maintained in all types of training, indicating that plasticity was not a short-term phenomenon, which was consistent with previous studies^36^. In terms of the far-transfer effect of daily living ability, no significant differences were found in the scores of the three groups, and these older adults had typical characteristics of those with MCI in this regard— their cognitive function was partially degraded, but the decline in their daily living ability was not obvious. Physical function, environmental factors, and cognitive function have important influences on daily living ability, but improvement is difficult to achieve with short-term cognitive training.

Most research on prospective memory emphasizes influencing factors and processing mechanisms^37^. This study selected prospective memory as an indicator of cognitive function and suggested a novel approach to help delay cognitive decline among older adults through the use of combined cognitive training which included prospective memory training. We found that the prospective memory ability of individuals with MCI was significantly improved after executive function training, indicating that the decline in prospective memory in older adults with MCI may be related to low executive function levels, which is consistent with the results of Wang et al.^22^. In terms of cognitive function, this study provides support for the view that executive function training can positively influence older adults’ cognitive function and provides strong experimental evidence for the executive decline hypothesis, which states that the specific decline of executive function is the main cause of cognitive aging. Currently, only a few studies have found cognitive plasticity in relation to inhibitory ability in older adults, and few studies have explored cognitive plasticity in individuals. This study found older adults with MCI, like healthy older adults, have executive function plasticity. However, the neuroplasticity of older adults with MCI was not examined in this study, and future research could explore changes in neural responses during training and their relationships with behavioral outcomes. When evaluating the effectiveness of cognitive training, the transfer effect of training should also be determined^38^. This study was undertaken with an awareness of the concept of “active aging” and aimed to provide further insights into treating AD.

This study had some limitations. First, this study only trained the “inhibition” function, and the process of completing the prospective memory task largely relied on conversion and inhibition of executive function^23^, which may be one reason why the training effect of combined cognitive training was not as significant as that of memory strategy training. Second, previous studies have found that individual differences influence the cognitive training effect and transfer effects^39^. In follow-up studies, researchers need to examine the benefits and transfer effects of training from the perspective of individual baseline levels.

## Conclusion

The conclusions of this study were as follows:

1. Compared to the control group, executive function training, memory strategy training, and combined cognitive training significantly improved prospective memory and executive function in older adults with MCI.
2. In terms of cognitive function, the improvement effect of combined cognitive training was significantly better than that of single executive function training and memory strategy training. In terms of prospective memory, the improvement effect of memory strategy training was superior to the other two kinds of training. In terms of daily living ability, none of the three training types showed an improvement effect.
3. Executive function training, memory strategy training, and combined cognitive training can maintain their training effect in older adults with MCI for three months.

## Methods

### Participants

This study recruited participants from five nursing homes in a city in China via convenience sampling. The inclusion criteria were—individuals aged 60 years and above with communicative and answering ability and with the help of community doctors, the participants with hearing, visual impairment, severe physical disabilities or weaknesses were excluded. Screening was conducted for those fitting the inclusion criteria who voluntarily participated in the study and for whom a clinical dementia rating (CDR) was obtained in cooperation with community doctors. According to Berg^40^, older adults with a CDR score of 0.5 are defined as older adults with MCI. In this study, 113 elderly individuals with MCI participated in the entire research process. They were randomly divided into a control group and three intervention groups, with 33 participants in the control group, 27 in the executive function training group, 26 in the memory strategy training group, and 27 in the combined cognitive training group. The details of the groups are listed in Table 1. The study was approved by the Ethics Committee of Ningbo University. When collecting information concerning the participants, their willingness to provide such information was respected. All individuals provided written informed consent in accordance with the Declaration of Helsinki. The participants were free to withdraw during the research process, and their privacy and personal information were respected and kept confidential. After the training, participants will be given some daily necessities, such as napkins, cooking oil and soap. The study protocol is performed in accordance with the relevant guidelines.

### Measurement tools

#### Go/No-Go tasks

The classic paradigm of inhibitory control training, Go/No-Go task testing, was adopted to train the participants’ executive function^41^. The training was conducted with E-prime 2.0, and the training duration was approximately 30 min each session. When the Go trial appeared on the screen, the participants were required to press a key, whereas when the No-Go trial appeared, no response was required. In the formal experiment, the computer screen presented a 1000 ms empty screen at first, and then a 1000 ms gaze point “ + ” was displayed in the center of the screen to prompt the participants to focus. Later, the target stimulus was presented at the center of the screen, requiring the participants to press a key. If they did not react in time, the screen would automatically skip to the next trial. There was a total of three blocks and 144 trials, with 48 trials in each block. After each training session, the background automatically recorded the accuracy and average response time of the participants: Go/No-Go task score = (accuracy/average response time) ^*^ 10,000. The higher the score, the better was the training effect.

### Stroop task

The Stroop task was used to train the participants’ conflict suppression ability^42^. The training was conducted through E-prime 2.0, and the training duration was approximately 30 min each session. In the formal experiment, the screen presented a 1000 ms empty screen at first, then a 1000 ms gaze point “ + ” was displayed in the center of the screen, prompting the participants to focus. Later, a target stimulus appeared in the center of the screen, requiring the participants to press a key to react. After the reaction, the screen automatically displayed the next trial. There was a total of three blocks, and 144 trials, with 48 trials in each block. After each training session, the background automatically recorded the accuracy and average response time of the participants: Stroop task score = (accuracy/average response time) ^*^ 10,000. The higher the score, the better was the training effect.

#### Fine encoding task

A fine encoding task was used to train the participants’ memory ability^43^. The training was conducted using E-prime 2.0 and included a total of three tasks. In task 1, the gaze point “ + ” was alternately presented with a total of 80 words, where the “ + ” was displayed at 3000 ms and the words displayed at 5000 ms. After confirming that the words were remembered, the participants reacted by pressing a key and then entered the next trial. In task 2, the participants identified whether an abstract or a concrete noun was being presented, with a total of 80 words. The gaze point “ + ” and the words appeared alternately, where the “ + ” was displayed at 3000 ms and the words displayed at 5000 ms. The participants had to press the “F” and “J” keys for the abstract and concrete nouns respectively. In task 3, the experimental materials comprised the 80 words in task 1, the 80 words in task 2, and 160 new words. The new and old words were presented randomly. The flow of each trial involved the gaze point “ + ” displayed at 3000 ms, with a random display of new or old words at 2775 ms, and then with the gaze point “ + ” displayed at 225 ms. Participants needed to respond to the new and old words by pressing the “F” and “J” keys for the new and old words respectively. After the key reaction was completed, the screen automatically displayed the next trial. The response time of task 1 was recorded as the score of task 1; the higher the score, the worse was the performance on task 1. The task scores of task 2 and task 3 were recorded as accuracy/average response time ^*^ 10,000, with higher scores indicating a higher level of the training’s effectiveness.

#### Montreal cognitive assessment scale

The Montreal Cognitive Assessment Scale (MoCA) compiled by Nasreddine et al.^44^ was used to assess cognitive ability among the older adults. The MoCA is an 11-item questionnaire that uses a 30-point Likert scale and includes eight dimensions—attention and concentration, executive function, memory, language, visual structure skills, abstract thinking, calculation, and orientation. A score of 18–25 indicates MCI, with a higher score indicating higher levels of cognitive ability^45,46^. In the current study, the Cronbach’s alpha coefficient was 0.82.

#### Instrumental activities of daily living scale

The Instrumental Activities of Daily Living Scale (IADL) compiled by Morrow et al.^47^ was used to assess the living ability of the participants. IADL is a self-evaluation scale. The IADL scale includes 7 dimensions of assessment: Entertainment and leisure, use of transport, cooking, housework, the ability to use the phone, take medication, the ability to manage money. Those with evaluation scores between 19–22 are considered competent, whereas those with evaluation scores between 15–18 are considered mildly disabled with higher scores indicating a better living ability^48,49^. In the current study, the Cronbach’s alpha coefficient of the scale was 0.87.

### Prospective and retrospective memory questionnaire (PRMQ)

The Chinese version of the Prospective and Retrospective Memory Questionnaire compiled by Crawford et al.^50^ was used to assess the prospective memory ability of the participants. It is a 16-item questionnaire that uses a five-point Likert scale ranging from 1 = “usually” to 5 = “never” with lower scores indicating a more severe damage to prospective and retrospective memory. In the current study, the Cronbach’s alpha coefficient of the scale was 0.91 (0.78, and 0.84 for prospective memory and retrospective memory, respectively). Given the research focus, only the prospective memory scale was used in the current study.

#### The virtual week (VW) task

The VW task compiled by Rendell and Carik^32^ was used to determine the prospective memory ability of the participants. The participants were required to roll a dice and move the corresponding number of squares on a chessboard. They also had to flip an event card indicating the day’s activities, such as eating. Each card had three choices. Taking the activity “reading books” as an example, the three choices were reading for 20, 30 min, or 40 min. After the participant had chosen, the event card would indicate a requirement in rolling the dice (such as rolling an even number). After the participants had understood the rules of the game, it began. One complete round of the board represented a day, and the participants had to complete seven rounds of the board and pick up 10 event cards in total. There were 122 squares on the board, representing the time from 7 am–10 pm. At the beginning of each round, each participant was required to obtain a 6 to represent waking up in the morning, after which the participant picked up a card, which would indicate the date of the day and two non-standard tasks that needed to be completed. When the participant returned to the “Start” position, this meant that the task for that round was complete, regardless of whether the dice number went beyond that square. The participants reported taking medication at 11 am and 9 pm every day, as well as after breakfast and dinner. When a clock showed 30 min twice each hour and for 15 min four times each hour, the participants had to check the time. In the formal experiment, each participant was required to conduct seven rounds of testing, with each round comprising 10 tasks and scoring 10 points. The average scores for the 10 tasks were recorded. The entire process took approximately 30 min to complete.

## Experimental design

A two-factor mixed experiment design of four intervention types (control group, executive function training, memory strategy training, and combined cognitive training) × 3 test times (pre-test, post-test, and follow-up) was used. The types of intervention comprised the inter-group variables, and the test times comprised the intra-group variables. The dependent variables were the changes in the participants’ test scores in terms of memory level, cognitive function, and daily living ability. The participants’ prospective memory level, cognitive function, and living ability were all tested in the pre-test, post-test, and follow-up phases. The scores on the MoCA, the IADL, the PRMQ-prospective memory (PRMQ-PM), and the VW task were taken as scores in relation to cognitive function, living ability and the level of prospective memory.

### Experimental process

**Figure 2:**
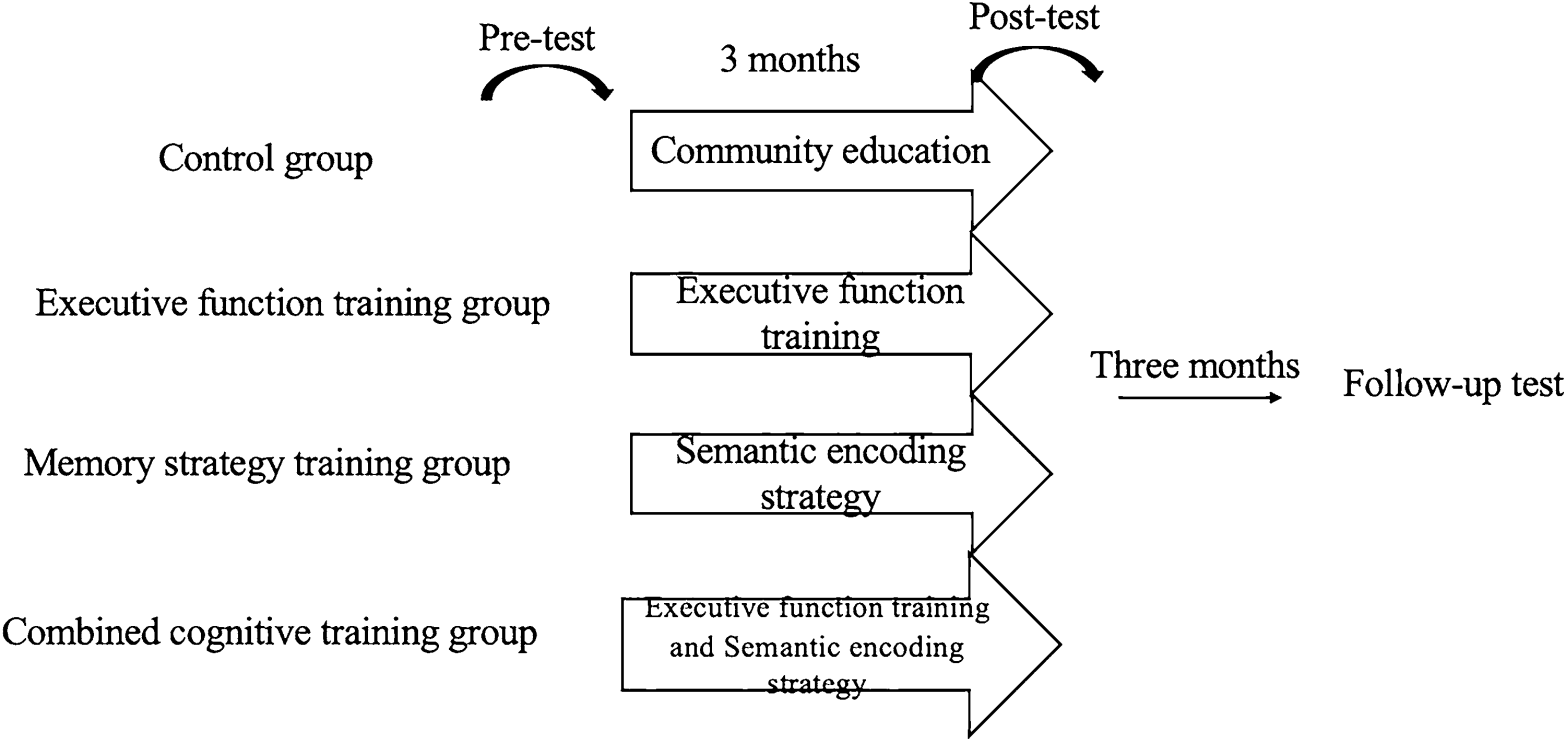
Experimental process.

The participants were randomly divided into four groups, namely control, executive function training, memory strategy training, and combined cognitive training groups. In the pre-tests, the participants’ MoCA, IADL, PRMQ-PM and VW task scores were recorded as their baseline level. Over three months, the control group received community education (Such as teaching health knowledge, smart phone use methods and other courses unrelated to the training content of the experimental group) twice a week for 30 min each session for a total of 24 training sessions. The executive function training group received executive function training twice a week (Go/No-Go, Stroop tasks) for 30 min each session for a total of 24 training sessions. The memory strategy training group received semantic encoding strategy training twice a week for 30 min each session for a total of 24 training sessions. The combined cognitive training group received executive functional training (Go/No-Go, Stroop) twice a week for 30 min each session for a total of 12 training sessions in the first six weeks. In the next six weeks, semantic encoding strategy training was conducted twice a week for 30 min each session for a total of 12 training sessions. In the current study, the training sequence used for the three intervention groups was to perform the Go/No-Go task first, followed by the Stroop task training, and finally the fine encoding training. At the end of each training session, the participants were given rewards or gifts. After training was completed, a post-test was conducted to compare the scores on the training tasks of each group, and to explore the transfer effect on prospective memory, cognitive function, and life ability. A follow-up test was conducted 3 months after training, in which the same content as the pre-test was tested (see Fig. 2).

### Analytic plan

One-way analyses of variance (ANOVA) were conducted to compare the baseline performance on cognitive tasks in four groups. A four-group (control group/executive function training group/memory strategy training group/combined cognitive training group) × 2 test time (pre-test/post-test) variance analysis of repeated measures were used to assess the training effects. A three group (executive function training group/memory strategy training group/combined cognitive training group) × 2 test time (pre-test/follow up-test) variance analysis of repeated measures were used to assess the maintenance effects.
